# pH Dependence of Succinimide-Ester-Based Protein Cross-Linking for Structural Mass Spectrometry Applications

**DOI:** 10.1021/acsmeasuresciau.1c00032

**Published:** 2021-11-11

**Authors:** Esben Trabjerg, Arend Keller, Alexander Leitner

**Affiliations:** Institute of Molecular Systems Biology, Department of Biology, ETH Zurich, 8093 Zurich, Switzerland

**Keywords:** Structural mass spectrometry, chemical cross-linking, disuccinimidyl suberate, pH dependence, specificity

## Abstract

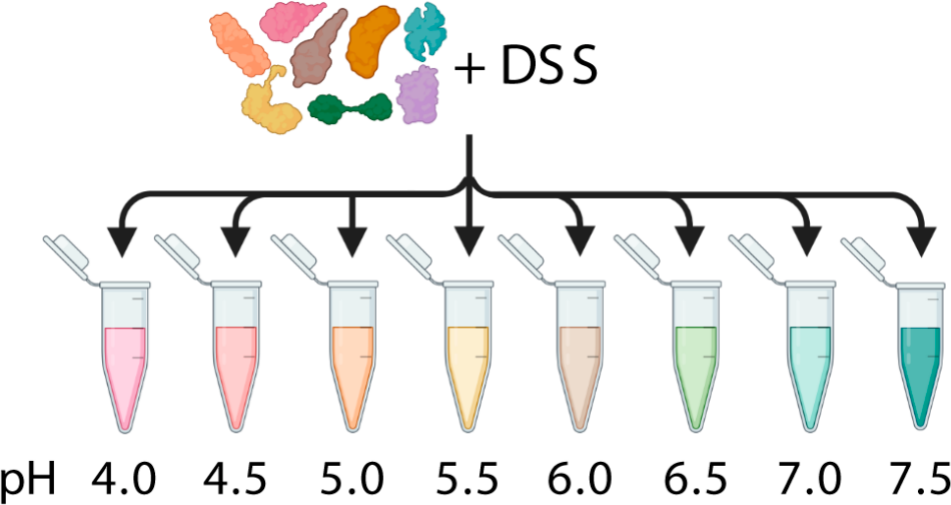

Within the research field of cross-linking mass spectrometry (XL-MS), the most commonly used cross-linking reagents are succinimide-ester-based (e.g., disuccinimidyl suberate (DSS)). These reagents primarily cross-link lysine side chains. So far, they have predominantly been used to investigate protein structures at neutral to slightly basic pH (7.0–8.5) to ensure the reactivity of the primary amine of the lysine side chain. However, disease-related molecular processes are not limited to such pH ranges; e.g., some important biological pathways are active in acidic intracellular compartments. The applicability of lysine-reactive cross-linking reagents to low-pH conditions remains unclear. Here, we cross-linked a mixture of eight model proteins at eight different pH conditions (pH 4.0–7.5) to investigate the pH dependency of DSS. DSS was able to cross-link proteins even at pH 4.0, but a clear decrease in the cross-linking efficiency was observed when the pH was lowered. Nevertheless, at pH 5.0, approximately half of the number of cross-links observed at pH 7.5 could still be identified. These findings highlight the ability of succinimide-based cross-linking reagents to be useful in probing the structure of proteins in a slightly acidic environment.

## Introduction

Cross-linking mass spectrometry (XL-MS) is a technique to delineate the structure of large proteins and protein complexes.^1−5^ In recent years, the method has been successfully applied to study large and challenging protein systems and has been shown to be highly powerful in combination with complementary structural biology methods, e.g., X-ray crystallography, cryogenic electron microscopy, and small-angle X-ray scattering.^1−4,6−10^

In the most common application of XL-MS, a protein or a protein complex of interest is treated with a chemical reagent that cross-links amino acid side chains under native conditions. After cross-linking and proteolytic cleavage, cross-linked peptides can be enriched by size exclusion, strong cation exchange chromatography, or affinity tags and separated by reverse-phase liquid chromatography before final mass analysis by high-resolution tandem MS (LC-MS/MS).^11−13^ Subsequently, the identity of the cross-linked residues and the length of the cross-linker can be used to provide “molecular rulers” that translate into atomic distance restraints for integrative molecular modeling of protein structures or large multiprotein complexes.^6,7,14−17^

**Figure 1:**
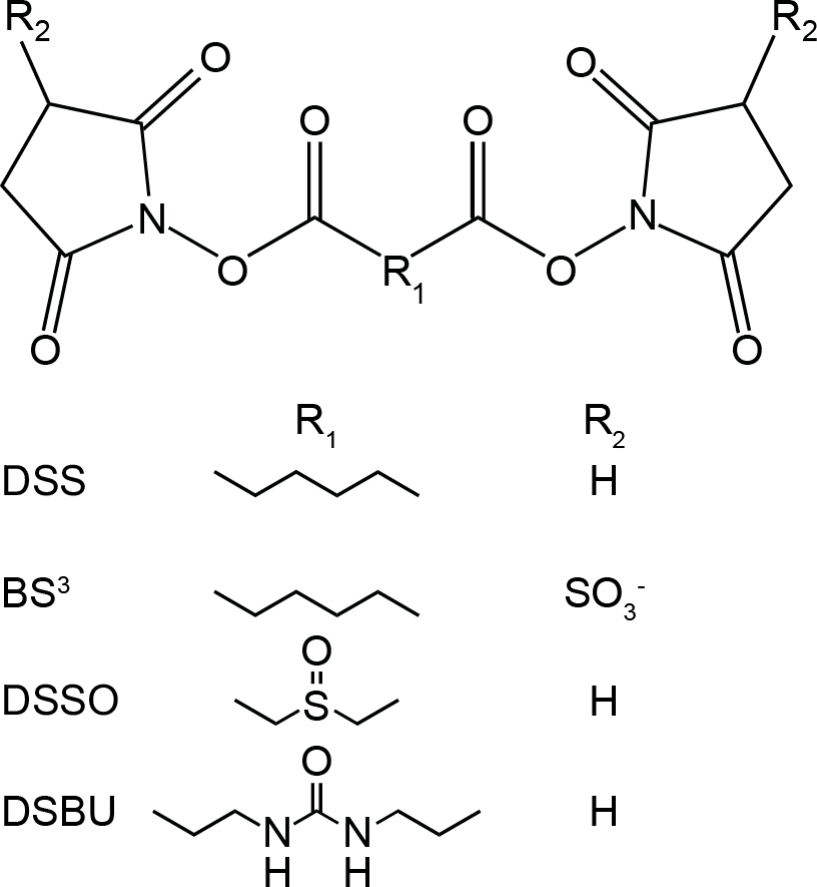
Chemical structures of NHS-based homobifunctional cross-linking reagents. All four cross-linking reagents are commercially available. DSS, BS^3^, and DSBU are available in isotopically labeled forms, and DSSO and DSBU are gas-phase cleavable.^23,25^

The most widely used cross-linking reagents are homobifunctional, *N*-hydroxysuccinimide (NHS)-based esters (e.g., disuccinimidyl suberate (DSS), bis(sulfosuccinimidyl) suberate (BS^3^), disuccinimidyl sulfoxide (DSSO), and disuccinimidyl dibutyric urea (DSBU)) (Figure 1).^18^ These reagents primarily connect primary amino groups (Lys residues and the N termini of proteins),^11^ but reactivity toward hydroxyl groups (Ser, Thr, and Tyr residues) has also been reported in the literature.^19−21^ Many NHS-based reagents are commercially available, including those that facilitate the reliable identification of cross-linked peptides through enhanced features, e.g., stable isotope labeling or gas-phase cleavable bonds.^22−25^

The cross-linking reaction for NHS esters is initiated by a nucleophilic attack from a free electron pair on a deprotonated primary amino group toward the NHS ester. To ensure the reactivity of the cross-linking reaction, the reaction has predominantly been performed at neutral to slightly basic conditions (pH 7.0–8.5).^11,26^ However, several important biological processes are not confined to neutral pH, as the pH in intracellular compartments spans several pH units from pH 8.0 in the mitochondria to pH 4.5–5.0 in lysosomes.^27,28^

Several contradictory reports have been published on the reactivity of NHS esters at slightly acidic conditions,^19,20,26^ but so far a comprehensive investigation of the reactivity of NHS-based cross-linkers for XL-MS applications has not been performed. To fill this gap, we performed a systematic evaluation of the cross-linking efficiency of DSS by targeting proteins in slightly acidic to neutral conditions (pH 4.0–7.5). Our results show a gradual but relatively moderate decrease in cross-linking efficiency with a decrease in pH: Approximately half of the cross-links identified at pH 7.5 are still observed under acidic conditions as low as pH 5.0.

## Results and Discussion

### Experimental Design

To assess the cross-linking efficiency of NHS-based reagents at slightly acidic conditions, we cross-linked a mixture of eight model proteins (bovine catalase, rabbit creatine kinase M-type, rabbit fructose-bisphosphate aldolase A, bovine serum albumin, chicken ovotransferrin, rabbit pyruvate kinase, bovine lactotransferrin, and bovine serotransferrin) at eight different pH conditions ranging from pH 4.0 to pH 7.5 with DSS. Together with its sulfonated analogue, BS^3^, DSS is the most widely used noncleavable cross-linking reagent. Here, we used a 1:1 mixture of nondeuterated and deuterated DSS, whereby in the deuterated version 12 hydrogen atoms in the spacer have been replaced by deuterium. This differential stable isotope labeling scheme generates a unique doublet signature for all peptides or peptide pairs that have reacted with the cross-linking reagent. The eight model proteins have previously been used to assess methodological improvements in XL-MS and the development of new cross-linking chemistries in our group.^11,29^ They are commercially available, they are of sufficient size relative to the distance restraint imposed by cross-linking (spanning a mass range of approximately 40–75 kDa), and their lysine content ranges between 5 and 10%. Moreover, the set contains both proteins that are monomeric and proteins that are homo-oligomers (dimers, tetramers) in their native state, while they do not interact with each other. In line with these expectations, the number of assigned interprotein cross-links was typically zero, and only for one pH step it reached three, which we consider false positives. Finally, the pH range was selected to cover the entire range from neutral/slightly basic to lower than what occurs naturally in lysosomes.

### The Specificity of DSS Is Not Affected by pH

**Figure 2:**
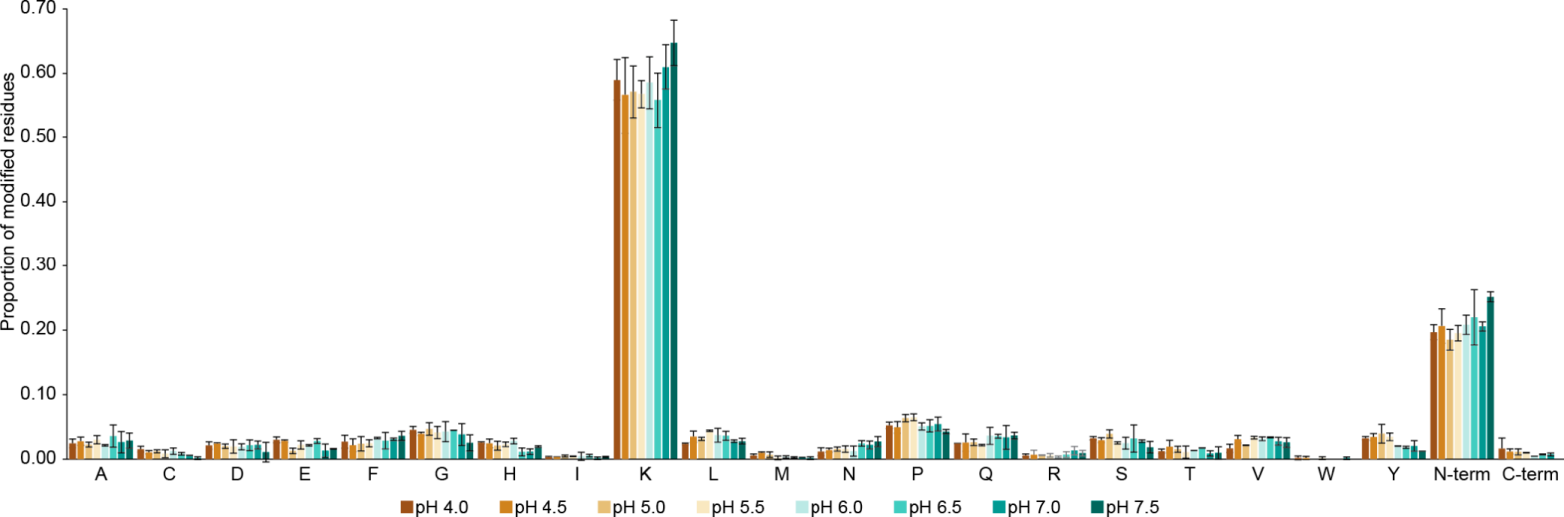
Residues labeled by hydrolyzed or amidated DSS moieties (mono-links/dead-end products, i.e., single peptide chains modified by the cross-linking reagent) as determined by MOD^a^.

It has previously been reported that the specificity of NHS ester cross-linking of proteins is affected by the pH of the reaction solution.^19,20^ To investigate the practical implications of such a pH dependency, we took advantage of the generation of mono-links (also described as “dead-end” products or “type 0” cross-links in the literature),^30^ where only one end of the cross-linker has reacted with a protein side chain. We performed a search for unrestricted modifications of peptides by the MOD^a^ algorithm.^31^ Here, peptides labeled by hydrolyzed or amidated DSS moieties (mono-links) could be easily identified due to the mass difference between differentially stable isotope-labeled DSS. As expected, Lys residues and protein N termini are the predominant targets of DSS (Figure 2).

Interestingly, the frequency of labeling of Ser, Thr, and Tyr residues was not notably higher than for other amino acids in our study. By carefully assessing the MOD^a^ data, it was obvious that most of the modifications not assigned to Lys residues or the N terminus might be attributed to inaccurate site localization of the DSS modification (Figure S1). Furthermore, the specificity of DSS is not markedly affected by the pH of the reaction solution. Therefore, the specificity of DSS toward Lys residues and the N terminus might decrease slightly below pH 7.0, but to an extent where it does not have any practical implications.

This is in contrast to a former study performed by Leavell et al.,^19^ where NHS acetate was used to label the model peptide, angiotensin I, and the oxidized β-chain of insulin. The authors observed a tendency of NHS esters to preferably label Tyr residues over Lys residues at pH 6.0, while Lys residues were favored at alkaline conditions (pH 8.4). A similar observation has been described by Swaim et al., who observed labeling of Ser and Tyr residues with an NHS-based cross-linker.^21^ In the current study, Lys residues and the N-terminal amino groups were preferentially labeled at all investigated pH conditions and the number of modifications at Tyr residues only appears to increase slightly below pH 6 (Figure 2). This discrepancy might be due to a difference in substrates between the two former studies and the current study. In the studies by Leavell et al. and Swaim et al., the investigated substrates were small and unstructured peptides, whereas large and structured proteins were used as substrates in our work. The presence of structure affects the microenvironment around the Lys side chains and modulates the p*K*_a_ of the lysine side chain, thereby potentially affecting its reactivity at lower pH conditions compared with a Lys residue side chain in an unstructured peptide.^32^ Furthermore, the reaction conditions differed markedly between the studies, as both Leavell et al. and Swaim et al. used a much higher cross-linker-to-substrate ratio compared to the current study.^19,21^ Most conventional XL-MS experiments use ratios between substrate and cross-linking reagents close to what has been employed in our experiments.^11,33^ Finally, the structural diversity of the substrates in the current study more closely reflects the targets commonly investigated by XL-MS compared to single proteins and unstructured peptides.

### DSS Is Able to Cross-Link Proteins at Slightly Acidic Conditions

To investigate the cross-linking efficiency of DSS at different pH conditions, the cross-linked protein mixture was analyzed by SDS-PAGE and digested with endoproteinase Lys-C and trypsin before analysis by reversed-phase nanoflow LC-MS/MS.^11^ SDS-PAGE showed a clear reduction in the cross-linking efficiency from pH 7.5 to pH 4.0. Furthermore, it is apparent that high-mass species are present for the two most acidic pH conditions (pH 4.0 and pH 4.5) (Figure S2). The presence of such cross-linked oligomers at low pH can be ascribed to the formation of aggregates that may have been present prior to or induced by the cross-linking reaction.

**Figure 3:**
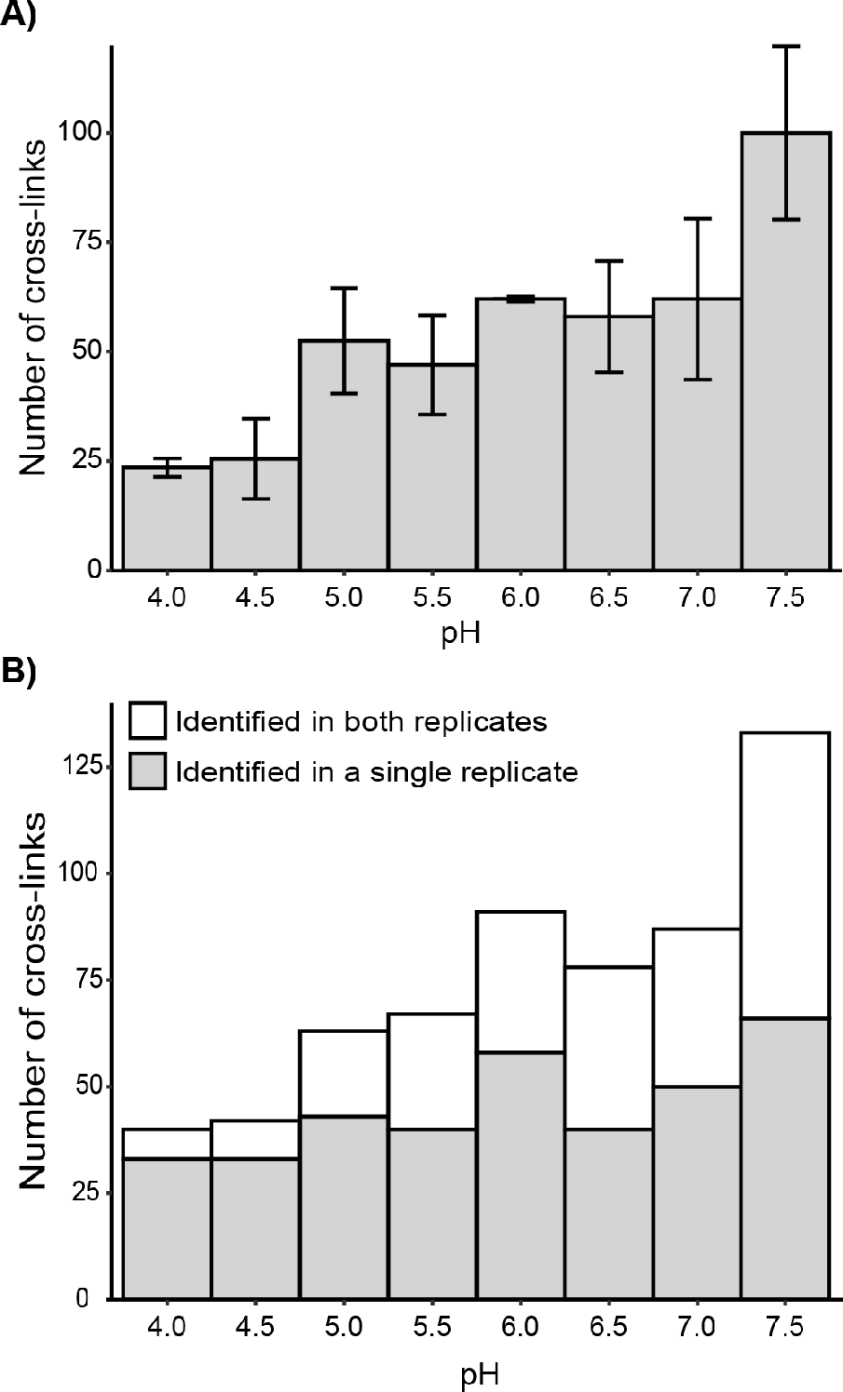
Identification of cross-linked peptide pairs at different pH conditions. (A) Number of cross-linked peptide pairs identified at different pH conditions. *n* = 2, error bars represent the standard deviation. (B) Reproducibility of the identified cross-linked peptide pairs from two experimental replicates. Gray, cross-linked peptide pairs identified in a single replicate; white, cross-linked peptide pairs identified in both replicates.

The LC-MS/MS data were searched by the dedicated search engine xQuest that is able to identify cross-linked peptide pairs by exploiting the presence of doublet signals separated by 12.0753 Da, corresponding to the mass difference between the two different isotopic variants of DSS. Intraprotein cross-linked peptide pairs could be identified over the entire pH range, but as expected the number of identified cross-links (unique peptide pairs) decreased with decreasing pH (Figure 3A, all identified cross-links are shown in Table S1), in line with the SDS-PAGE results. By decreasing the pH from 7.5 to 5.5, a 2-fold decrease in the number of identified cross-links was observed, while a further 2-fold decrease in the number of identified cross-links was observed when lowering the pH all the way down to pH 4.0 (the most acidic condition investigated in the current study).

Instead of investigating the numbers of identified cross-links in the two replicate experiments, we merged the results to investigate if the reproducibility of the experiments was constant over the investigated pH range (Figure 3B). Here, it is evident that the reproducibility decreases markedly at lower pH. At pH 7.5, approximately half of the cross-linked peptide pairs are identified in both replicates (white section of the bars), while this is below one-fifth of the cross-linked residue pairs at pH 4.0. This observation is probably due to a decrease in the intensity of cross-linking products; hence, fewer of them were chosen for fragmentation in each replicate injection, as the mass spectrometer was operated in data-dependent acquisition mode.

The drop in the number of identified cross-linked peptide pairs at lower pH conditions is much lower than what could be theorized from the deprotonation of Lys side chains caused by the drop in pH. A 1.5 unit drop in pH (e.g., from pH 7.5 to 6.0), will cause a 31-fold decrease in the presence of deprotonated Lys side chains (Supporting Information). However, such a corresponding drop in the amount of identified cross-links was not observed. This can be due to several factors. First, a relatively large excess of NHS ester was used in the current study, resulting in a high chance for a Lys side chain to react with DSS, even if only a small fraction of the amino group is in the deprotonated state. Second, the hydrolysis rate of DSS in an aqueous environment is decreased at acidic conditions, which increases the active concentration of reactive DSS throughout the incubation time.^26,34^

### The Distance Distribution of Identified Cross-Links Is Not Affected by pH

**Figure 4:**
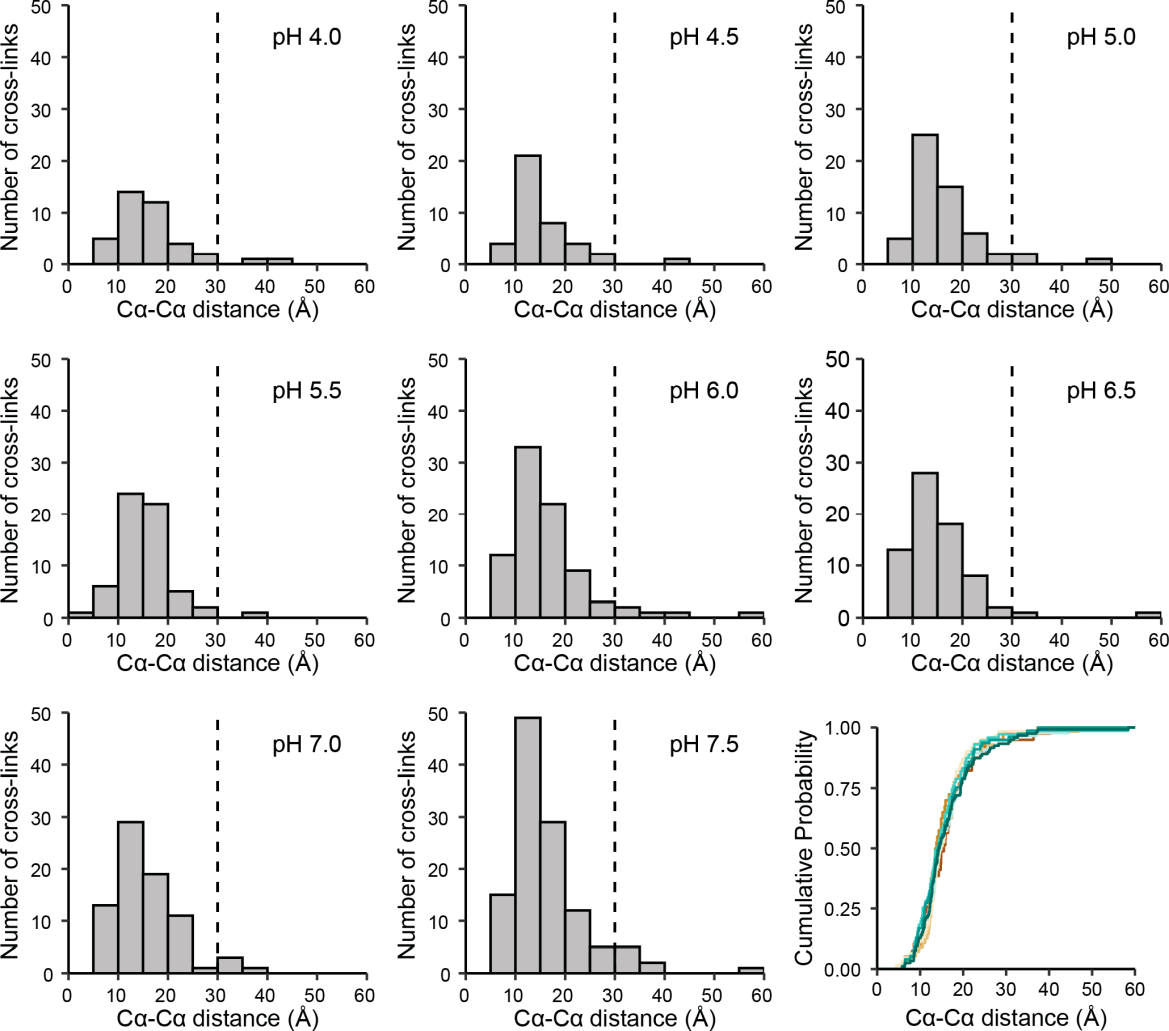
Distance distribution of identified unique cross-linked residue pairs. Euclidian Cα–Cα distances of unique cross-linked residue pairs (site pairs) identified in the eight investigated pH conditions (pH 4.0–pH 7.5). The black dotted line (30 Å) marks the commonly used distance cutoff empirically determined for DSS.^11^ The panel in the bottom right corner depicts the cumulative distribution of distances. The same color scheme as in Figure 2 is used.

The purpose of many XL-MS applications on proteins and protein complexes is to generate distance constraints/restraints, which subsequently can be used for structural interpretation such as integrative structural modeling. Hence, we investigated the distance distribution of all the identified cross-link pairs identified at the different pH conditions (Euclidean Cα–Cα distances) (Figure 4). Here, it is evident that no marked difference in the distance distribution was observed in the investigated pH range. Furthermore, the number of cross-linked residue pairs exceeding 30 Å, which corresponds to an approximate upper bound for DSS, is quite stable over the investigated pH range (Table S2). Even though signs of pH-induced aggregation at the lower pH conditions were observed (Figure S2), these aggregates do not seem to be efficiently cross-linked or result in insoluble aggregates that are not digested by LysC and trypsin.

We also looked at the distance distributions for individual proteins (Figures S3–S11). Although the number of cross-linked peptide pairs per protein differed widely (Figure S3), the numbers decrease with decreasing pH or remain relatively stable. None of the proteins showed a clear tendency for observing cross-links exceeding 30 Å at low pH when matched to 3D structures (Figures S4–S11).

The results presented herein reflect the ability of DSS to produce structural information on proteins at slightly acidic conditions and expand the applicable pH window of DSS. In the literature, NHS esters have mainly been used to investigate protein structures at neutral to slightly basic pH, to ensure reactivity.^11,26^ A previous study by Mädler et al. performed on unstructured peptides showed the inability of DSS to react with lysine-containing peptides at pH 6.1 at shorter incubation times (20 min).^20^ In support of our findings is the early work by Cuatrecasas and Parikh, who successfully coupled NHS esters and primary amines in the pH range from pH 6.0 to pH 9.0.^34^ The observed discrepancy between the results obtained in the current study and the observations described by Mädler et al. can be due to several different factors. First of all, the detection limit for observing cross-linked peptide products by LC-MS/MS has decreased considerably in the past decade due to improvements in instrumentation and software.^1^ Furthermore, as alluded to earlier, the current study was performed on proteins with a complex, three-dimensional fold, at least at neutral pH. An organized three-dimensional arrangement affects the microenvironment surrounding lysine residues and thereby modifies their p*K*_a_ and will affect their reactivity toward DSS.^32^

Quite likely, the described trends are not limited to DSS but are also applicable to other NHS-based cross-linking reagents, e.g., BS^3^, DSSO, and DSBU, although this needs to be confirmed experimentally.

## Conclusion

In conclusion, the presented results highlight the potential of DSS to cross-link proteins not only at neutral to slightly basic conditions but also at slightly acidic conditions. This widens the use and applicability of DSS for tackling the structural characterization of proteins and protein complexes found at acidic conditions, e.g., molecular machines in acidic intracellular compartments or protein complexes implicated in viral entry into a host cell.

## Experimental Section

### Materials

All reagents, including the model proteins, were purchased from Sigma-Aldrich as analytical grade except the following: DSS (*d*_0_/*d*_12_) (Creative Molecules Inc., Canada), MS grade trypsin (Promega Corporation, USA), and Lys-C (Fujifilm Wako Pure Chemical Corp., Japan).

### Cross-Linking Reaction

Initially, stock solutions of eight proteins (bovine catalase (UniProt ID P00432), rabbit creatine kinase M-type (UniProt ID P00563), rabbit fructose-bisphosphate aldolase A (UniProt ID P00883), bovine serum albumin (UniProt ID P02769), chicken ovotransferrin (UniProt ID P02789), rabbit pyruvate kinase (UniProt ID P11974), bovine lactotransferrin (UniProt ID P24627), and bovine serotransferrin (UniProt ID Q29443)) were prepared in phosphate-buffered saline at concentrations of 5 mg/mL. Stock solutions were then combined in equal proportions of proteins per weight and the protein mixture was diluted to 2 mg/mL total protein concentration (0.25 mg/mL per protein, corresponding to approximately 3–6 μM) with a citrate-phosphate buffer to obtain the desired pH. The citric acid/phosphate buffer was obtained by mixing a 0.1 M citric acid solution with a 0.2 M Na_2_HPO_4_ solution according to the scheme developed by McIlvaine^35^ (for details, see Table S3). A total of eight pH conditions were investigated in duplicate (pH 4.0, 4.5, 5.0, 5.5, 6.0, 6.5, 7.0, and 7.5). After addition of the citric-phosphate buffer, the sample was left at 25 °C for at least 30 min before the addition of DSS. For each cross-linking experiment, 50 μg of protein at a total protein concentration of 2 mg/mL was cross-linked with 1 mM DSS (*d*_0_/*d*_12_) for 30 min at 37 °C. The reaction was quenched by the addition of NH_4_HCO_3_ to a final concentration of 50 mM.

### Digestion and Solid-Phase-Extraction Cleanup

The samples were digested by Lys-C and trypsin proteases and purified by solid-phase extraction as described previously.^11,12^ In short, the cross-linked protein mixture was dried in a vacuum centrifuge, resuspended in 8 M urea to a concentration of 1 mg/mL protein, reduced (2.5 mM tris(2-carboxyethyl)phosphine HCl), and alkylated in the dark (5 mM iodoacetamide). Afterward, the protein mixture was digested with a two-step digestion procedure with Lys-C (1:100 enzyme/substrate) and trypsin (1:50). After an overnight digestion, the samples were acidified with formic acid (2%) and purified by solid-phase-extraction (Sep-Pak 50 mg tC18 cartridges, Waters, USA). The eluate (water/acetonitrile/formic acid, 50:50:0.1 v/v) was evaporated to dryness and resuspended in solvent A (water/acetonitrile/formic acid, 95:5:0.1 v/v).

### Liquid Chromatography-Tandem Mass Spectrometry

An amount of 1.25 μg of peptides was directly loaded onto an Acclaim PepMap column (150 mm × 75 μm, 2 μm particle size, 100 Å pore size, ThermoFisher Scientific, USA) in a nanoflow LC system (EASY-nLC 1000, ThermoFisher Scientific, USA). The peptide mixture was separated by a 120 min gradient from 9% to 35% solvent B (water/acetonitrile/formic acid, 98:2:0.15, v/v/v). The peptides were ionized by positive-mode electrospray ionization and analyzed in a hybrid ion trap-orbitrap mass spectrometer (Orbitrap Elite, ThermoFisher Scientific, USA). The instrument was operated in data-dependent acquisition mode, where the survey scan was performed in the orbitrap (350–1600 *m*/*z*) with a resolution of 120 000. The 10 most abundant ions with a charge state ≥ 3 were fragmented by collision-induced dissociation in the ion trap with 35% normalized collision energy. The *m*/*z* of the resulting fragment ions were determined in the linear ion trap (200–2000 *m*/*z*). All samples were analyzed in technical duplicate.

### Data Analysis

#### MOD^a^ Analysis

The raw files of the MS/MS data were converted into MGF format by msconvert (ProteoWizard, version 3.0.9393)^36^ and searched by the MOD^a^ software, version 1.60.^31^ The following settings were used: Instrument = ESI-TRAP; PeptTolerance = 0.02 Da; FragTolerance = 0.6 Da; BlindMode = 1 (one modification per peptide); ModSize = [−50;+300] (mass range for modifications in Da); Enzyme = trypsin, MissedCleavage = 2. The protein database contained the eight standard proteins and their shuffled decoys. The results were further analyzed using the anal_moda.jar script, and only identifications with a false discovery rate < 0.01 were kept for further analysis. The resulting matrix of mass shifts was filtered for paired mass shifts of 12 Da.

#### xQuest Analysis

The raw files of the MS/MS data were converted into the mzXML format by msconvert (ProteoWizard).^36^ Cross-linked peptides were identified by xQuest (version 2.1.5).^37,38^ The combination of the heavy and light scans was performed with the following settings: Precursor mass difference: 12.07532 Da, retention time difference for light/heavy pairs: 1.0 min. The database contained the sequence of all eight standard proteins. For identification of cross-linked peptides, the following settings were applied: Maximum number of missed cleavages (excluding the cross-linking site) = 2, peptide length = 4–40 amino acids, fixed modifications = carbamidomethyl-Cys (mass shift = 57.02146 Da), mass shift of the light cross-linker = 138.06808, mass shift of mono-links = 155.09463 or 156.07864 Da, MS1 tolerance = 15 ppm, and MS2 tolerance = 0.2 Da for common ions and 0.3 for cross-link ions; the search was performed in ion tag mode. The analysis was performed at the level of unique cross-link peptide pairs, and only cross-linking hits with a score ≥ 25 were considered for further interpretation and included in the further data analysis performed in RStudio (RStudio Inc., USA) and Excel (Microsoft Corp., USA).

### Data Availability

The mass spectrometry raw files have been deposited to the ProteomeXchange Consortium via the PRIDE partner repository with the data set identifier PXD027891.^39^ All other data are available from the corresponding author upon reasonable request.

## Supporting Information Available

Over-length cross-linked residue pairs identified at the different pH conditions; preparation of the buffer solutions; specificity of DSS at different pH conditions; SDS-PAGE gel of the cross-linking reaction of the eight model proteins cross-linked at eight different pH conditions; details about cross-links on individual proteins and their distance distributions; relative decrease in the amount of deprotonated lysine side chains at pH 6.0 compared to pH 7.5 (PDF)
Summary of all cross-link identifications (XLSX)
