# Supplementary material for: pH Dependence of Succinimide-Ester-Based Protein Cross-Linking for Structural Mass Spectrometry Applications
Source: ACS Meas Sci Au. 2021 Nov 11;2(2):132–8. doi: 10.1021/acsmeasuresciau.1c00032 (PMC9838815; doi:10.1021/acsmeasuresciau.1c00032)
Supplement: Supplementary file 1 — tg1c00032_si_001.pdf [file tg1c00032_si_001.pdf]

Supporting Information to

pH dependence of succinimide ester-based protein cross-linking for structural mass spectrometry applications

Esben Trabjerg, Arend Keller and Alexander Leitner*

Institute of Molecular Systems Biology, Department of Biology, ETH Zurich, Switzerland

* To whom correspondence should be addressed.

Table of Contents

Page S3: **Table S2.** Over-length cross-linked residue pairs identified at the different pH conditions.

Page S4: **Table S3.** Overview of preparation of the pH buffers.

Page S5: **Figure S1.** Specificity of DSS at different pH conditions.

Page S6: **Figure S2**. SDS-PAGE gel of the cross-linking reaction of the eight model proteins cross-linked at eight different pH conditions.

Page S7: **Figure S3**. Identification of cross-linked peptide pairs at different pH conditions for the eight single investigated proteins.

Page S8: **Figure S4**. Distance distribution of identified unique cross-linked residue pairs from bovine catalase at different pH conditions.

Page S9: **Figure S5**. Distance distribution of identified unique cross-linked residue pairs from rabbit creatine kinase M-type at different pH conditions.

Page S10: **Figure S6**. Distance distribution of identified unique cross-linked residue pairs from rabbit fructose-bisphosphate aldolase A at different pH conditions.

Page S11: **Figure S7**. Distance distribution of identified unique cross-linked residue pairs from bovine serum albumin at different pH conditions.

Page S12: **Figure S8**. Distance distribution of identified unique cross-linked residue pairs from chicken ovotransferrin at different pH conditions.

Page S13: **Figure S9**. Distance distribution of identified unique cross-linked residue pairs from rabbit pyruvate kinase at different pH conditions.

Page S14: **Figure S10**. Distance distribution of identified unique cross-linked residue pairs from bovine lactotransferrin at different pH conditions.

Page S15: **Figure S11**. Distance distribution of identified unique cross-linked residue pairs from bovine serotransferrin at different pH conditions.

Page S16: Relative decrease in the amount of deprotonated lysine side chains at pH 6.0 compared to pH 7.5.

Provided as a separate file in Microsoft Excel format: **Table S1.** Summary of all cross-link identifications.

Table S2. Over-length cross-linked residue pairs identified at the different pH conditions.

**pH**	**4.0**	**4.5**	**5.0**	**5.5**	**6.0**	**6.5**	**7.0**	**7.5**
**Unique over length cross-link pairs (>30Å)**	2	1	3	1	5	2	4	8
**Unique cross link pairs**	39	40	56	61	84	73	78	119
**Percentage over length cross-link pairs (>30Å) (%)**	5.13	2.25	5.36	1.64	5.95	2.74	5.13	6.72

Table S3. Overview of preparation of the pH buffers. To obtain 10.00 ml of the respective pH buffers solutions of 0.1 M citric acid and 0.2 M Na_2HPO_4 were mixed according to the table below. If necessary, the pH was fine-tuned with either 0.1 M citric acid or 0.2 M Na_2HPO_4 to obtain the desired pH.

pH	0.1 M citric acid (mL)	0.2 M Na_2HPO_4 (mL)
**4.0**	6.145	3.855
**4.5**	5.457	4.543
**5.0**	4.85	5.15
**5.5**	4.312	5.688
**6.0**	3.685	6.315
**6.5**	2.9	7.1
**7.0**	1.765	8.235
**7.5**	0.775	9.225
**8.0**	0.275	9.725

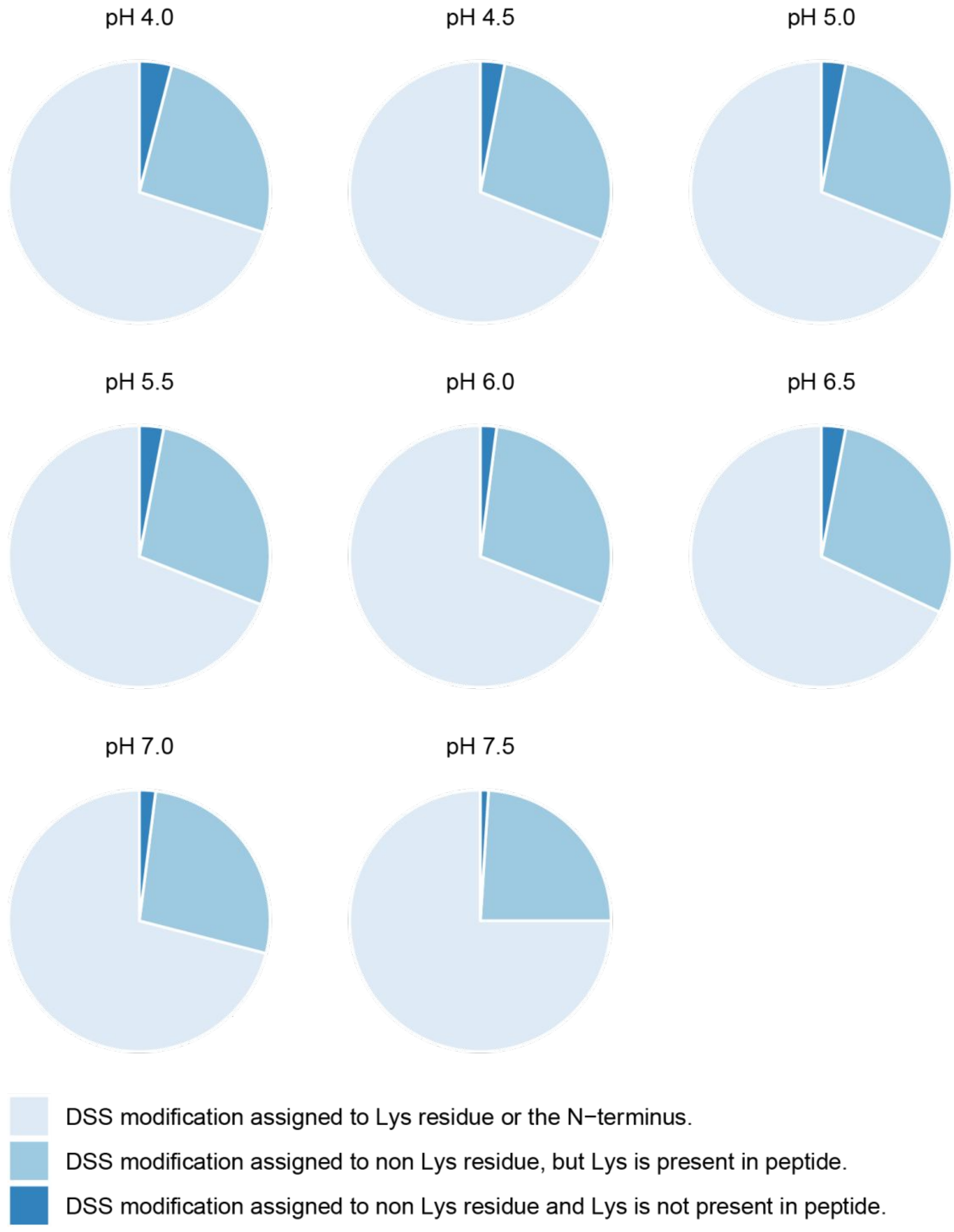

Figure S1. Specificity of DSS investigated by the MOD[a] algorithm. The peptides identified as modified by DSS with MOD[a] were binned into three different categories: 1) Peptides for which the DSS modification was assigned to a Lys residue or the N-terminus; 2) Peptides for which the DSS modification was not assigned to a Lys residue or the N-terminus, but the peptide contained a Lys residue; and 3) Peptides for which the DSS modification was not assigned to a Lys residue or the N-terminus, and the peptides did not contain a Lys residue.

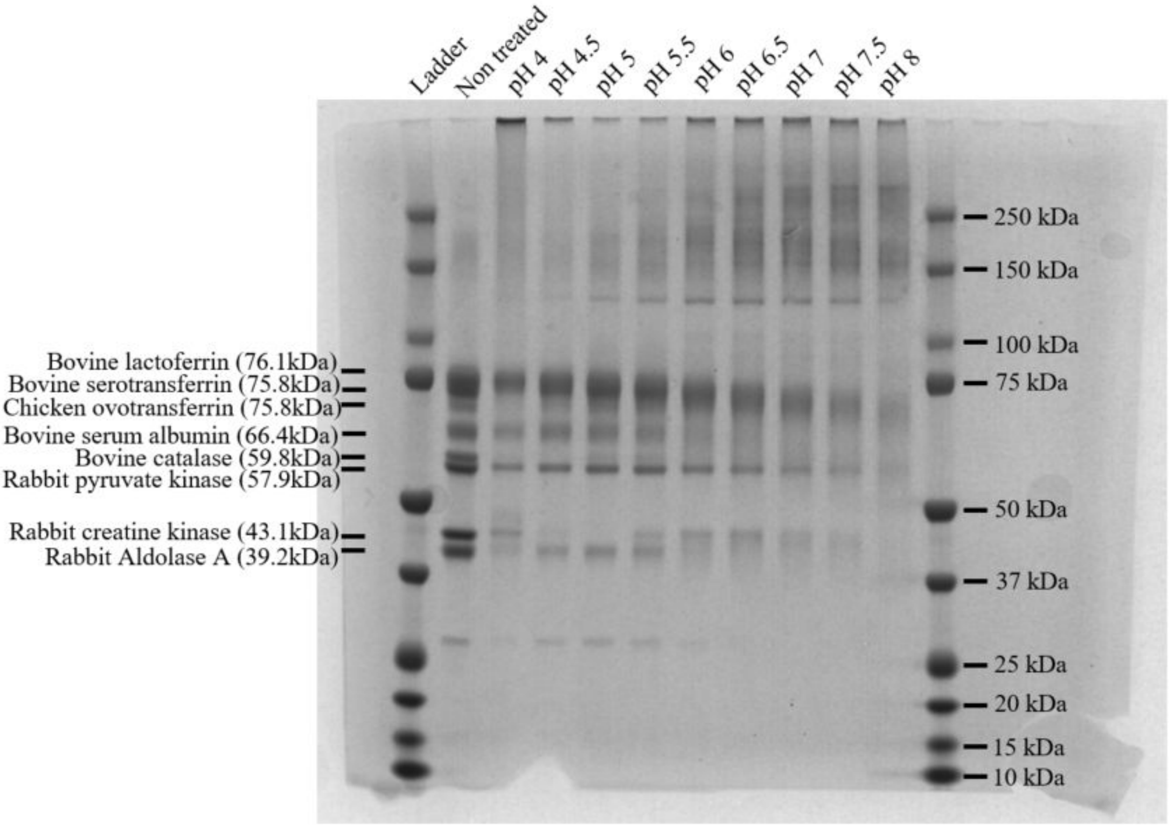

Figure S2. SDS-PAGE gel of the cross-linking reaction of the eight model proteins cross-linked at eight different pH conditions.

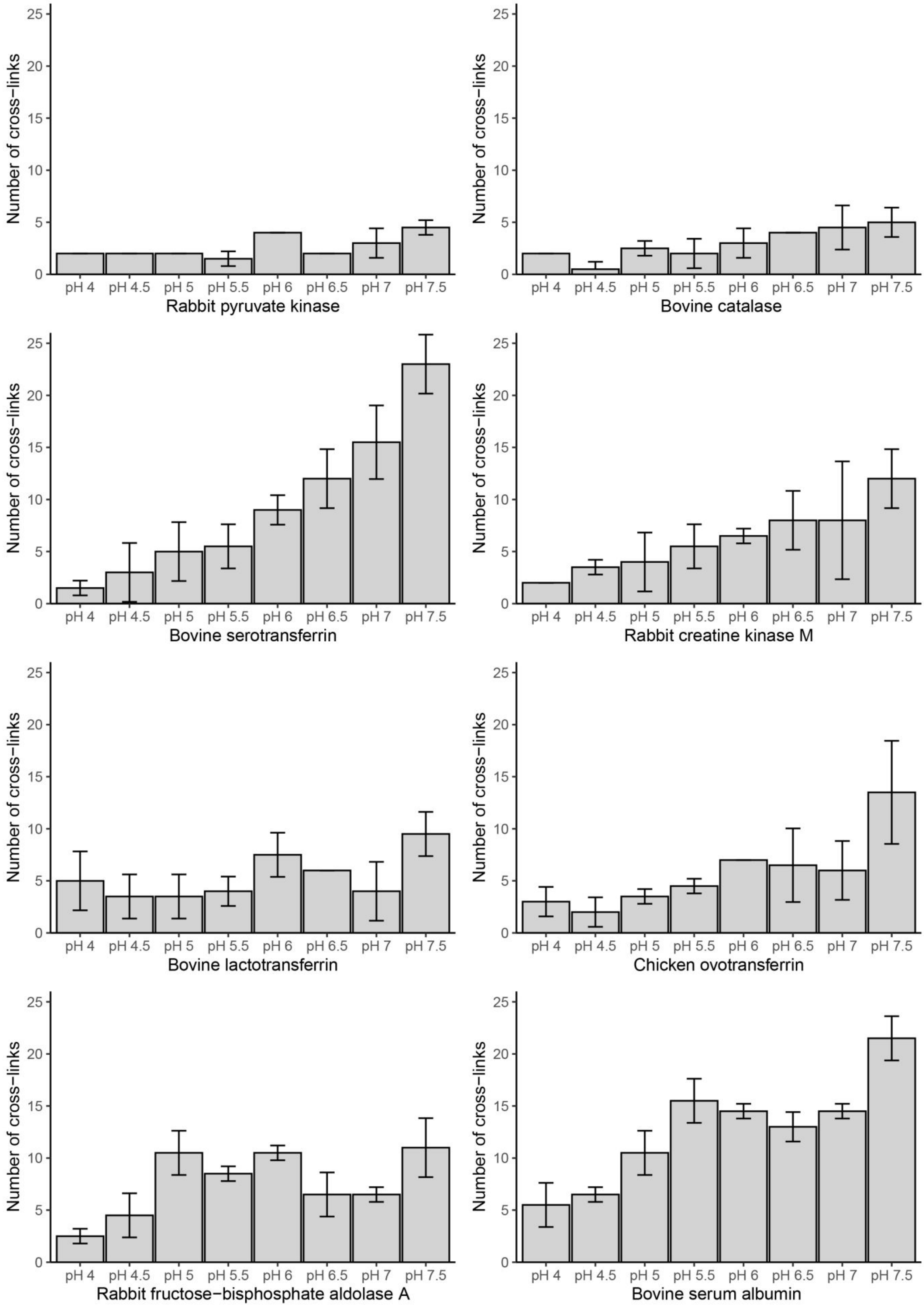

Figure S3. Identification of unique cross-linked peptide pairs at different pH conditions for the eight investigated proteins.

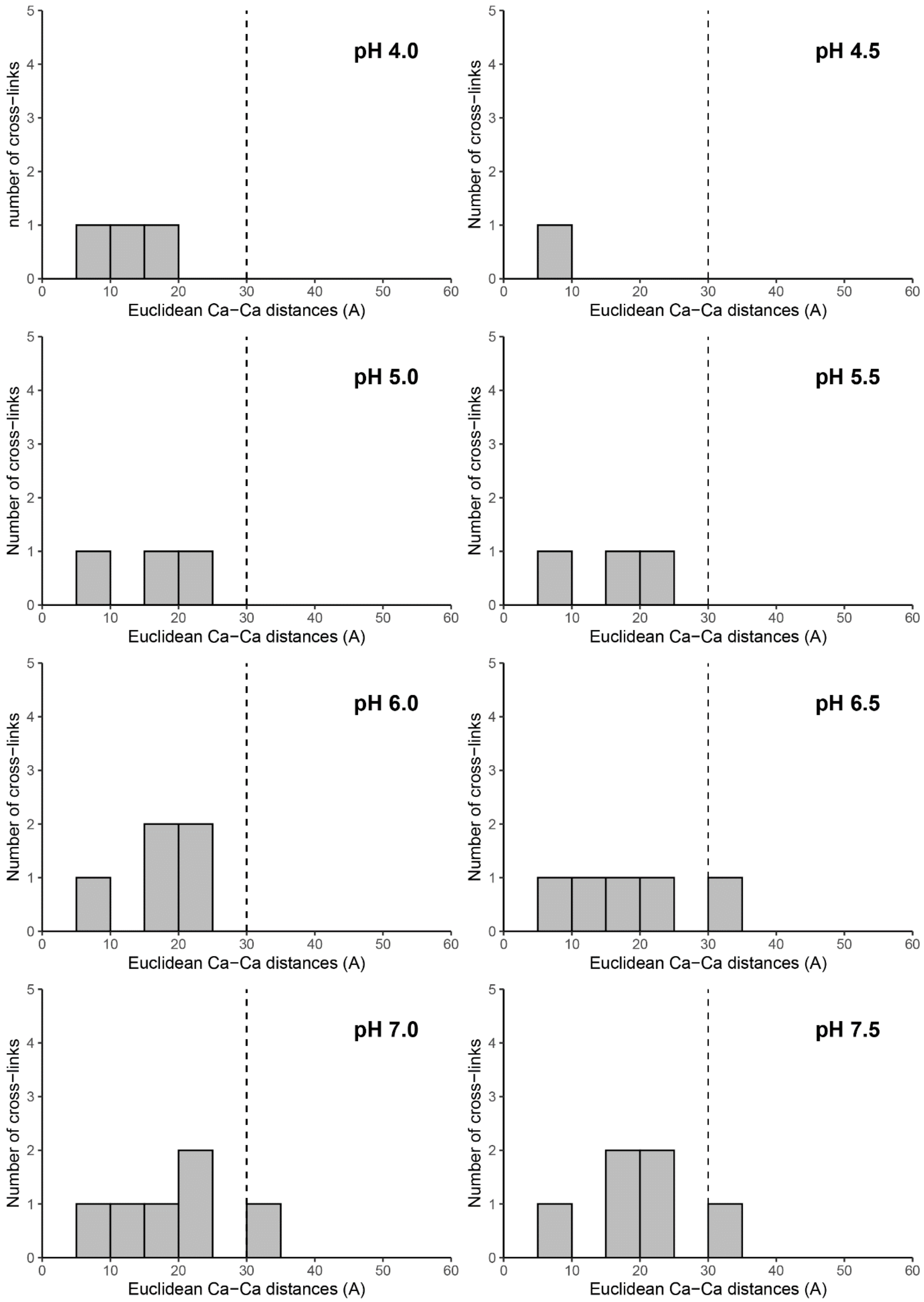

Figure S4. Distance distribution of identified unique cross-linked residue pairs from bovine catalase at different pH conditions. The black dotted line (30 Å) marks the commonly used distance cutoff empirically determined for DSS.

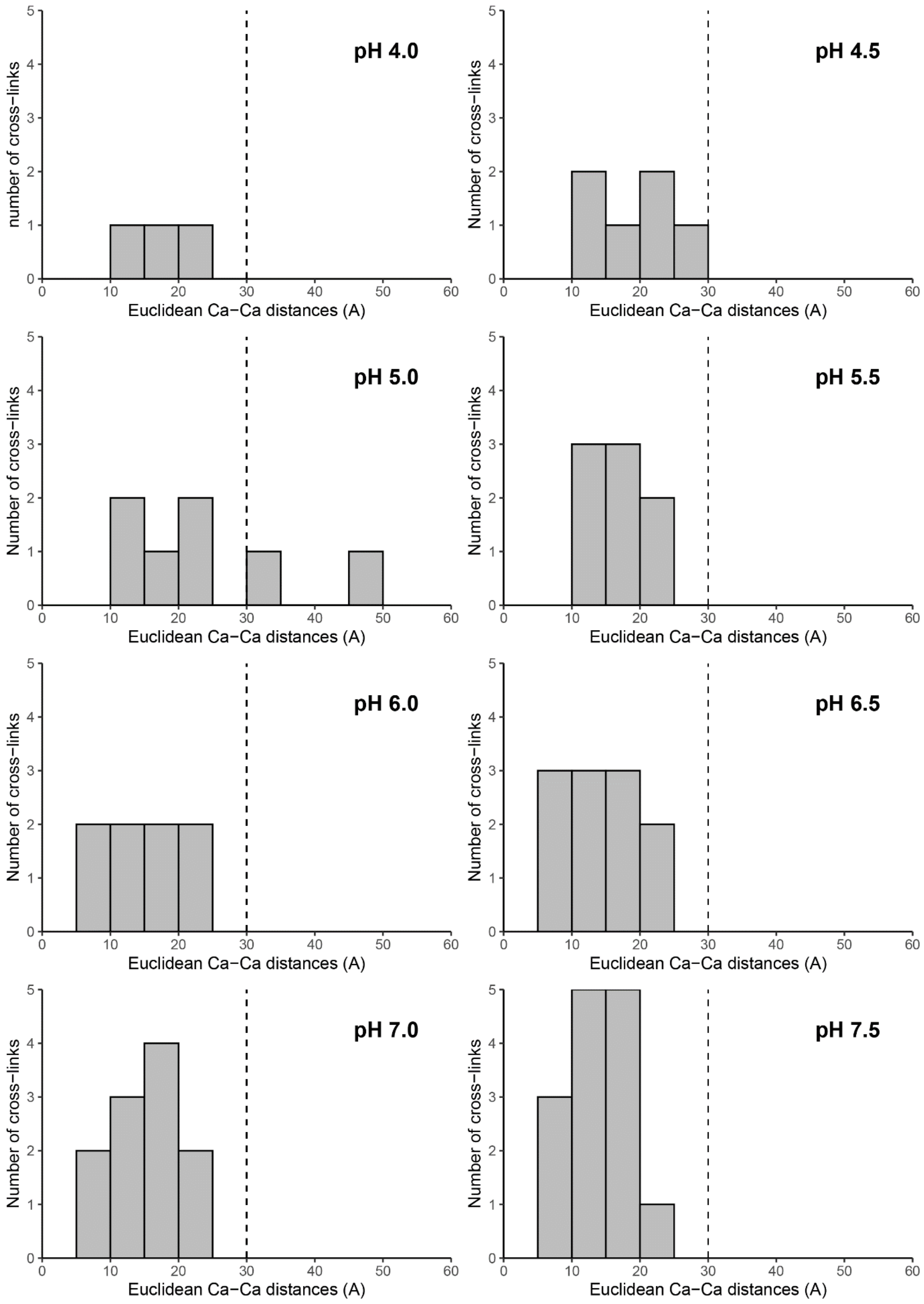

Figure S5. Distance distribution of identified unique cross-linked residue pairs from rabbit creatine kinase M-type at different pH conditions. The black dotted line (30 Å) marks the commonly used distance cutoff empirically determined for DSS.

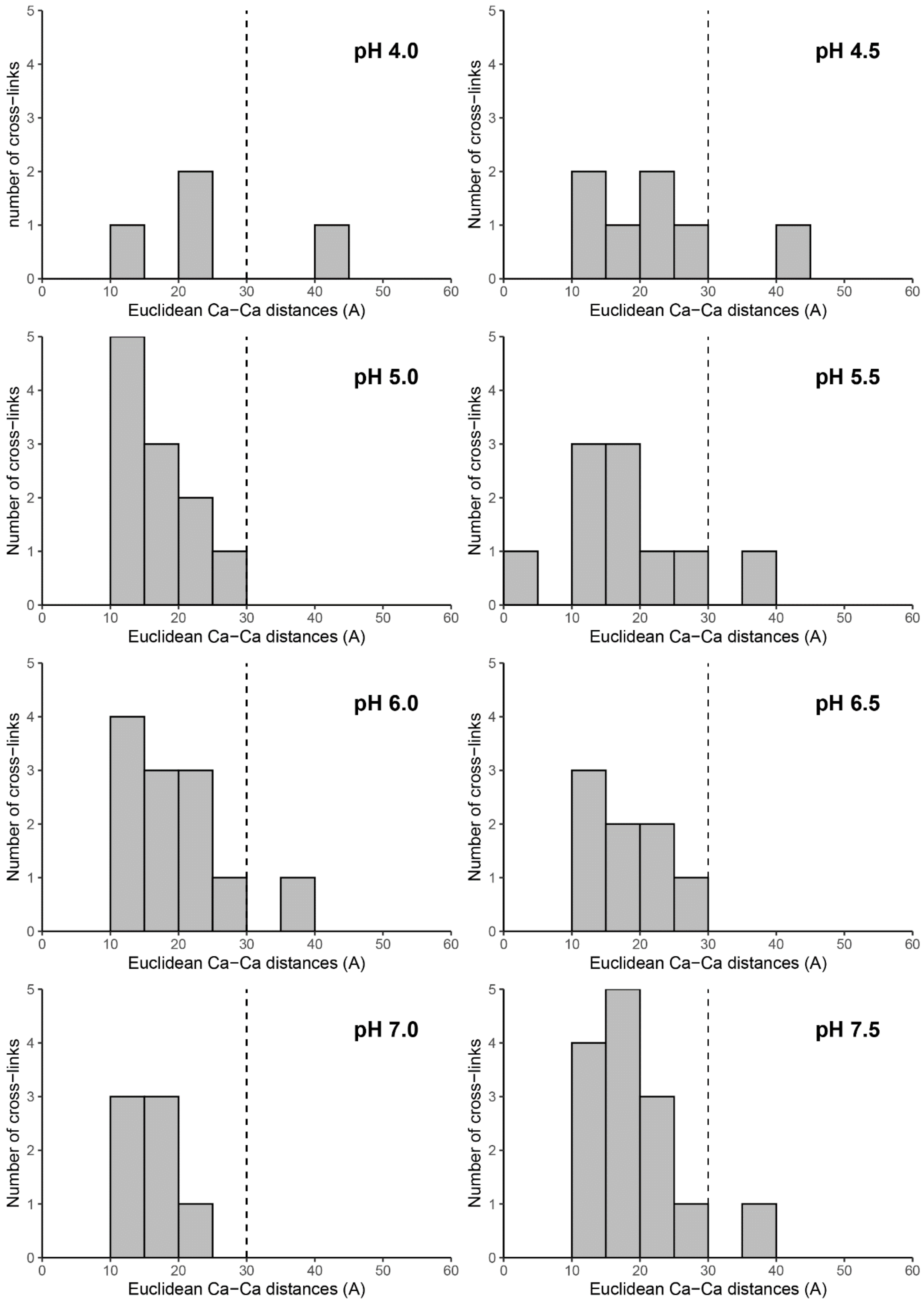

Figure S6. Distance distribution of identified unique cross-linked residue pairs from rabbit fructose-bisphosphate aldolase A at different pH conditions. The black dotted line (30 Å) marks the commonly used distance cutoff empirically determined for DSS.

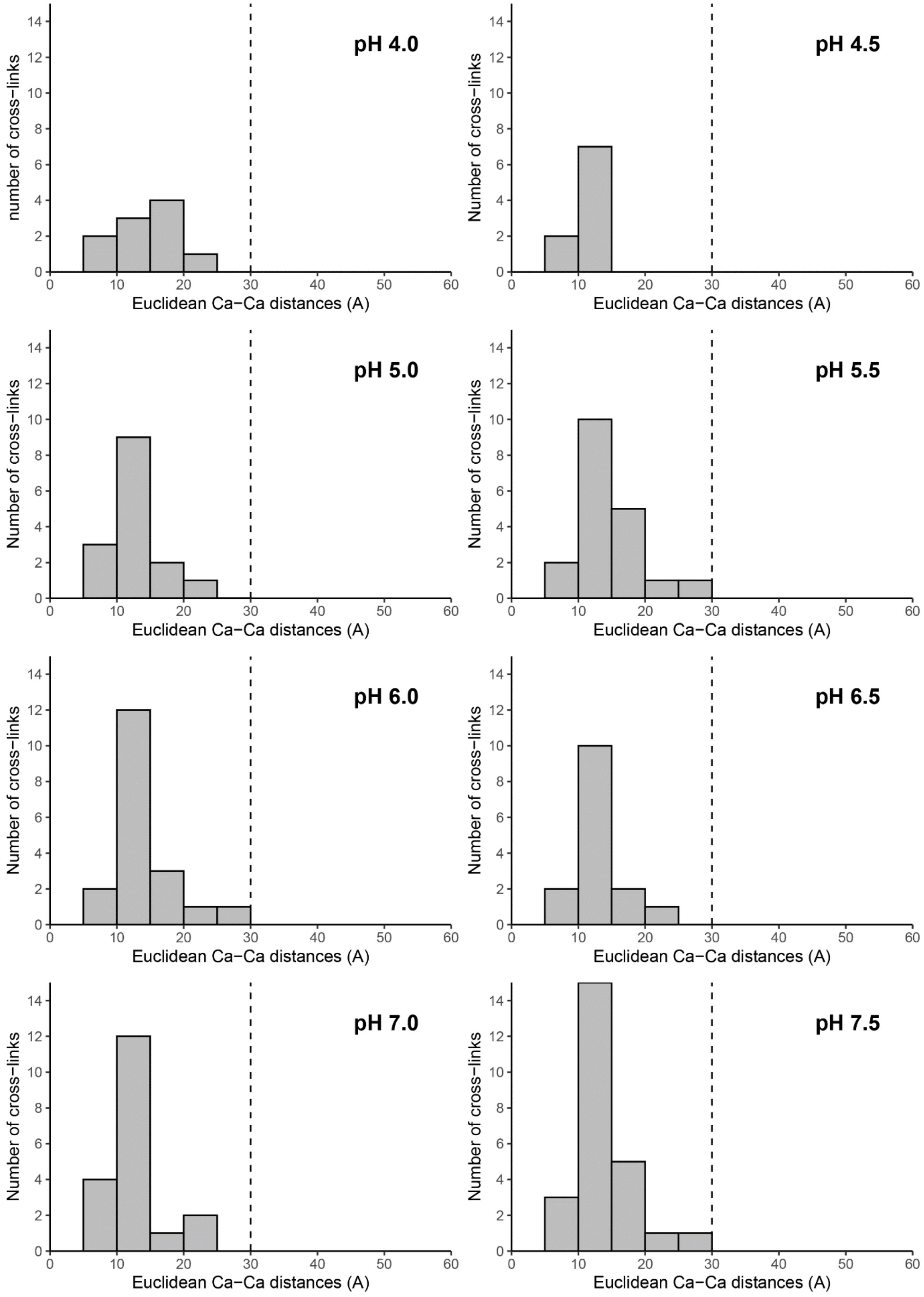

Figure S7. Distance distribution of identified unique cross-linked residue pairs from bovine serum albumin at different pH conditions. The black dotted line (30 Å) marks the commonly used distance cutoff empirically determined for DSS.

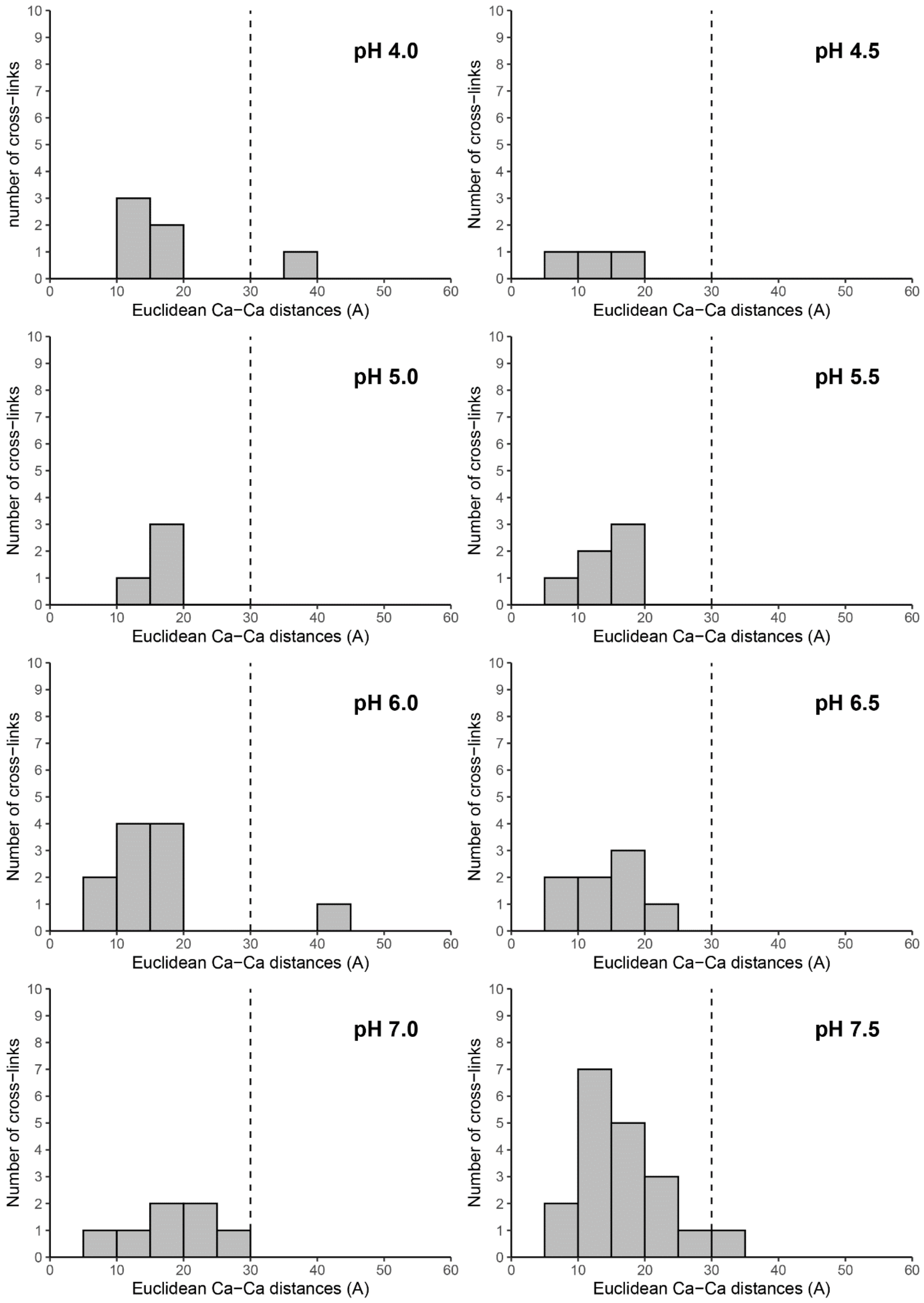

Figure S8. Distance distribution of identified unique cross-linked residue pairs from chicken ovotransferrin at different pH conditions. The black dotted line (30 Å) marks the commonly used distance cutoff empirically determined for DSS.

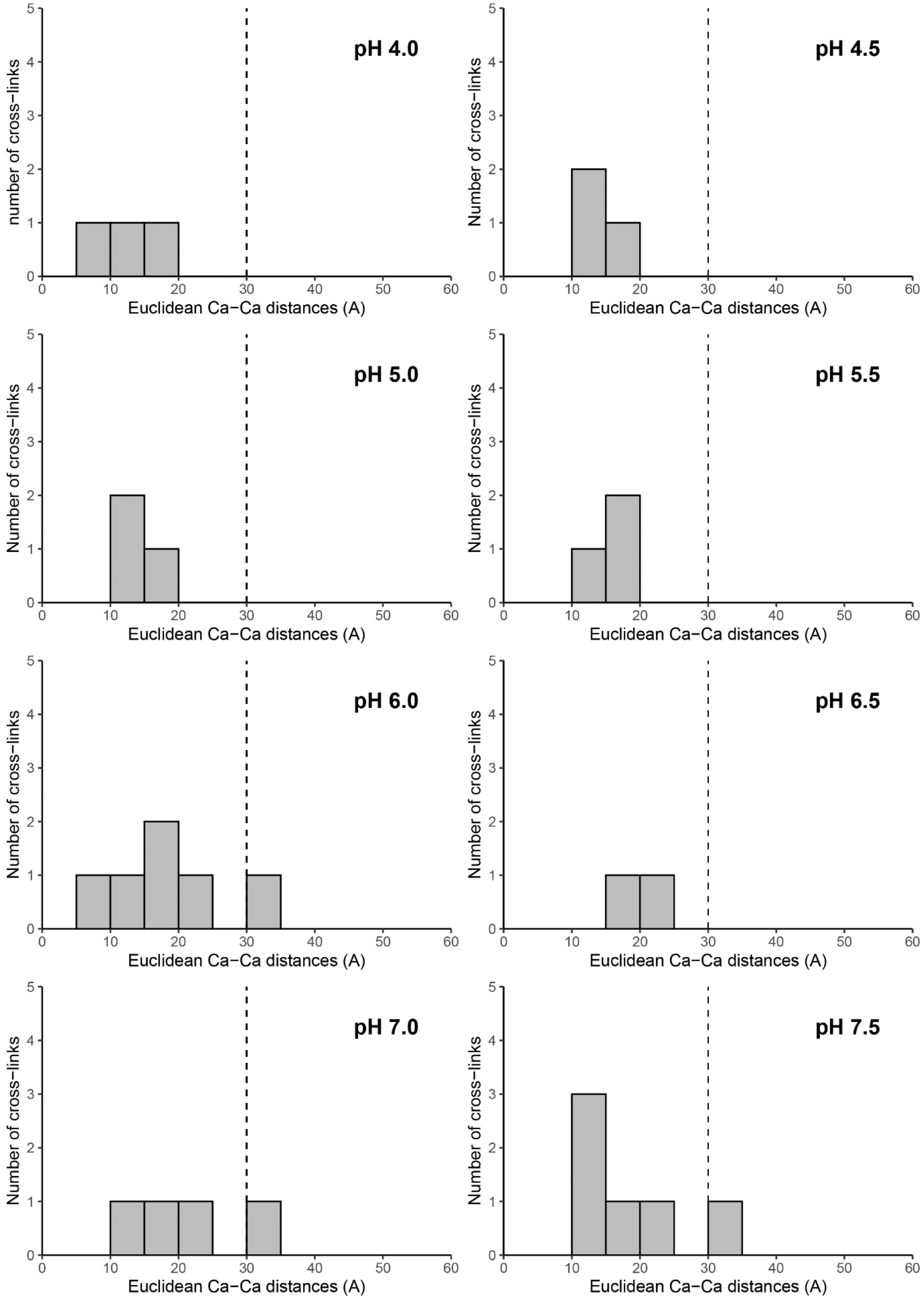

Figure S9. Distance distribution of identified unique cross-linked residue pairs from rabbit pyruvate kinase at different pH conditions. The black dotted line (30 Å) marks the commonly used distance cutoff empirically determined for DSS.

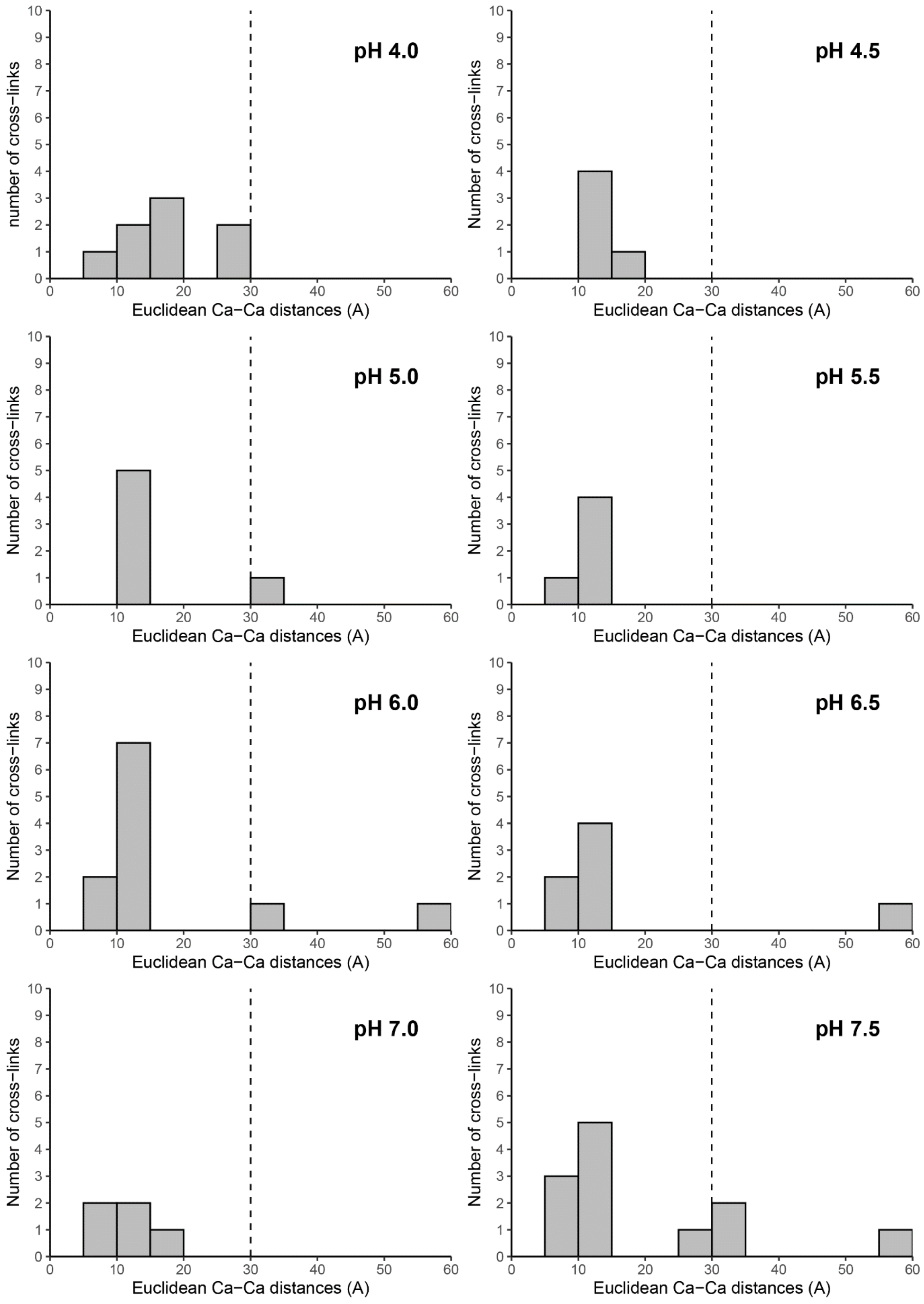

Figure S10. Distance distribution of identified unique cross-linked residue pairs from bovine lactotransferrin at different pH conditions. The black dotted line (30 Å) marks the commonly used distance cutoff empirically determined for DSS.

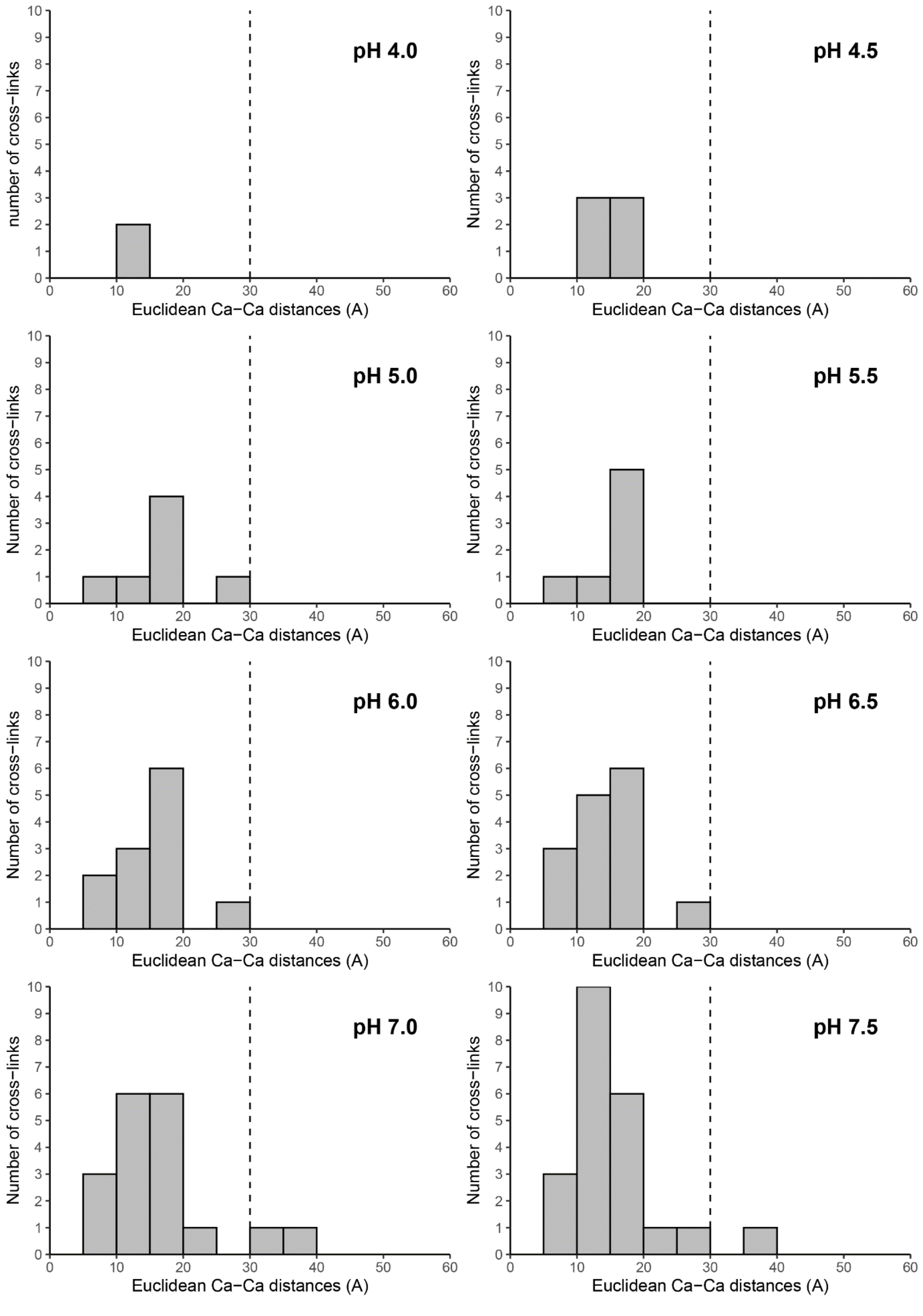

Figure S11. Distance distribution of identified unique cross-linked residue pairs from bovine serotransferrin at different pH conditions. The black dotted line (30 Å) marks the commonly used distance cutoff empirically determined for DSS.

Relative decrease of the amount of deprotonated lysine side chains at pH 6.0 compared to pH 7.5

The first step in the DSS cross-linking reaction of two lysine residues is the nucleophilic attack of a free electron pair on a non-protonated lysine side chain upon the NHS ester:

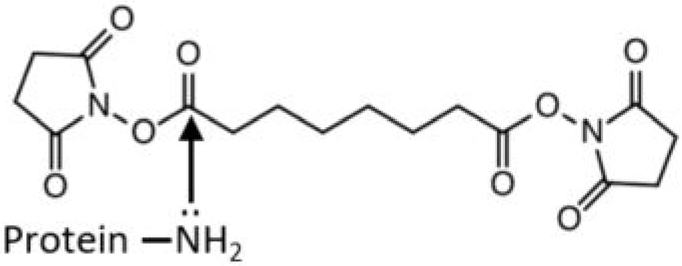

The relative proportion of lysine side chains that are deprotonated at a specific pH condition can be estimated by the Henderson-Hasselbach equation:

$$pH = pK_a + \log\left(\frac{[R-NH_2]}{[R-NH_3^+]}\right),$$

Where the pK_a of the lysine side chain is 10.82.*

The relationship between the deprotonated and protonated form of the lysine side chain at pH 6.00 and pH 7.50 can now be estimated.

At pH 6.00:

$$6.00 = 10.82 + \log\left(\frac{[R-NH_2]}{[R-NH_3^+]}\right) \Leftrightarrow \frac{[R-NH_2]}{[R-NH_3^+]} = 1.51 \times 10^{-5}$$

At pH 7.50:

$$7.5 = 10.82 + \log\left(\frac{[R-NH_2]}{[R-NH_3^+]}\right) \Leftrightarrow \frac{[R-NH_2]}{[R-NH_3^+]} = 4.79 \times 10^{-4}$$

The fold decrease of the amount of deprotonated lysine side chains can now be estimated from pH 7.5 to 6.0:

$$fold\ decrease = \frac{4.79 \times 10^{-4}}{1.51 \times 10^{-5}} = 31.6$$

* Harris, D.C.: Quantitative Chemical Analysis. W. H. Freeman and Company, New York, USA (2010)

Similar calculations can be performed for comparison of all investigated pH conditions:

Decrease in pH	Fold decrease in the amount of deprotonated lysine side chains
0.5	3.2
1.0	10.0
1.5	31.6
2.0	100.0
2.5	316.2
3.0	1000.0
3.5	3162.3
